# RNA-Seq study reveals genetic responses of diverse wild soybean accessions to increased ozone levels

**DOI:** 10.1186/s12864-017-3876-2

**Published:** 2017-06-29

**Authors:** Nathan Waldeck, Kent Burkey, Thomas Carter, David Dickey, Qijian Song, Earl Taliercio

**Affiliations:** 1 Driskill Graduate Program in Life Sciences, Northwestern University Feinberg School of Medicine, Chicago, IL 60611 USA; 2 Soybean and Nitrogen Fixation Research Unit, and the Department of Crop and Soil Sciences, North Carolina State University, Raleigh, NC 27695 USA; 3 USDA/ARS, Soybean and Nitrogen Fixation Unit, Raleigh, NC 27695 USA; 4 Statistics Department, North Carolina State University, Raleigh, NC 27695 USA; 5 USDA/ARS, Soybean Genomics and Improvement Laboratory, BARC-West, Beltsville, MD 20705 USA

**Keywords:** Abiotic stress, Defense, Photosynthesis, Wild soybean germplasm

## Abstract

**Background:**

Ozone is an air pollutant widely known to cause a decrease in productivity in many plant species, including soybean (*Glycine max* (L.) Merr). While the response of cultivated soybean to ozone has been studied, very little information is available regarding the ozone response of its wild relatives.

**Results:**

Ozone-resistant wild soybean accessions were identified by measuring the response of a genetically diverse group of 66 wild soybean (*Glycine soja* Zucc. and Sieb.) accessions to elevated ozone levels. RNA-Seq analyses were performed on leaves of different ages from selected ozone-sensitive and ozone-resistant accessions that were subjected to treatment with an environmentally relevant level of ozone. Many more genes responded to elevated ozone in the two ozone-sensitive accessions than in the ozone-resistant accessions. Analyses of the ozone response genes indicated that leaves of different ages responded differently to ozone. Older leaves displayed a consistent reduction in expression of genes involved in photosynthesis in response to ozone, while changes in expression of defense genes dominated younger leaf tissue in response to ozone. As expected, there is a substantial difference between the response of ozone-sensitive and ozone-resistant accessions. Genes associated with photosystem 2 were substantially reduced in expression in response to ozone in the ozone-resistant accessions. A decrease in peptidase inhibitors was one of several responses specific to one of the ozone resistant accessions.

**Conclusion:**

The decrease in expression in genes associated with photosynthesis confirms that the photosynthetic apparatus may be an early casualty in response to moderate levels of ozone. A compromise of photosynthesis would substantially impact plant growth and seed production. However, the resistant accessions may preserve their photosynthetic apparatus in response to the ozone levels used in this study. Older leaf tissue of the ozone-resistant accessions showed a unique down-regulation of genes associated with endopeptidase inhibitor activity. This study demonstrates the existence of significant diversity in wild soybean for ozone response. Wild soybean accessions characterized in this study can be used by soybean breeders to enhance ozone tolerance of this important food crop.

**Electronic supplementary material:**

The online version of this article (doi:10.1186/s12864-017-3876-2) contains supplementary material, which is available to authorized users.

## Background

The deleterious effects of ozone (O_3_) on agriculture have been of increasing concern. Ozone levels have risen from <10 parts per billion (ppb) in the late 1800s [1] to daytime levels of nearly 40 ppb in many areas of the Northern Hemisphere [2]. As populations rise and technology advances, it is expected that these levels could increase to 75 ppb by the year 2050 [3]. Increased O_3_ levels have proven detrimental to plant health; the damages to carbon assimilation, stomatal conductance, and plant growth result in significant losses to crop yield [4–7]. Losses in total crop global production have been estimated to be from $14 billion to $26 billion [8].

Ozone reacts with many different molecules in the apoplast of leaves to produce different harmful products known as Reactive Oxygen Species (ROS), such as OH^−^ radicals and hydrogen peroxide [9, 10]. Therefore, the capacity of the apoplast to detoxify ROS is a primary defense mechanism of the plant to ozone stress [11]. After exposure to O_3_ levels of >40 ppb, stress responses of sensitive plants are mediated through changes in ROS, phytohormone levels, calcium ion binding and changes in mitogen-activated protein kinase (MAPK) signaling cascades. The O_3_ response pathway shares many similarities to that of programmed cell death often seen in response to pathogens, and both stresses are associated with characteristic lesions [12]. Both processes involve a rise in endogenous ROS production in response to stress, which further activates the ethylene, salicylic acid, and jasmonic acid pathways for expression of defense genes [13]. This also emphasizes the dual role of ROS, both as a signaling molecule and potential toxin.

Ozone injury occurs across a continuum from chronic to acute effects ranging from physiological changes (e.g. accelerated senescence) to various types of foliar symptoms described as purple stippling, bronzing, chlorotic mottling, or leaf flecking that begin as small lesions and can develop into large areas of necrosis. The injury observed may vary from chronic to acute depending upon the atmospheric O_3_ concentration, duration of the exposure, environmental factors affecting stomatal conductance (and thus O_3_ uptake), and the inherent O_3_ sensitivity of the plant. Ultimately, this injury decreases photosynthesis resulting in reduced plant biomass and yield [7]. Stomatal sensitivity to abscisic acid may also be affected by O_3_ stress, causing transpiration levels to increase when exposed to drought [14]. Lower stomatal conductance is likely to indicate a decrease in photosynthesis [15].

Recent studies on *Glycine max* (L.) Merr. showed a reduction of up to 25% in yield after O_3_ exposure largely due to smaller seed size and fewer pods per plant [16]. This is consistent with other studies showing reduced photosynthesis in soybean as well as in other crops such as wheat and rice [17, 6, 4]. Increased cellular respiration due to ozone stress may lead to a further reduction in productivity [18].

Burkey and Carter [19] evaluated 30 North American ancestral soybean lines to determine if O_3_ resistance exists in the background of present cultivars. A ten-fold difference was discovered between responses in these lines indicating that the development of resistant soybean varieties is possible using current germplasm. Whaley et al. [20] conducted a follow-up gene expression analysis of a resistant *G. max* cultivar and North American ancestor, Fiskeby III, and a sensitive ancestor, Mandarin (Ottawa) subjected to treatment with high [75 ppb] and low [25 ppb] ozone. This study showed that cultivated soybean exhibits differential expression among expected ROS and defense pathways, but there are cultivar-specific gene differences between resistant and sensitive cultivars. Expression of many genes varied substantially at different time points for the sensitive cultivar with different stress response genes responding at different times. In contrast, the resistant soybean cultivar showed a consistent defense response and an up-regulation in metabolism genes. The Whaley study also found a possible link between leaf architecture and O_3_ response as demonstrated by the increased expression of wax and cutin biosynthesis genes of the sensitive variety. In addition, Burton et al. [21] identified several putative QTL for O_3_ resistance in a mapping population created from a cross between Mandarin (Ottawa) and Fiskeby III. In the same study, a different response between younger and more mature leaves was noted.

The aim of these experiments is to identify novel sources of O_3_ resistance for soybean and gain insight into the physiological/molecular basis of that resistance. In this study, we characterize the O_3_ response of 66 wild soybean (*Glycine soja* Zucc and Sieb) accessions representative of the genetic diversity in the USDA soybean collection to identify O_3_ resistant accessions. We also characterize the gene expression response of two O_3_-sensitive accessions and two O_3_-resistant accessions using RNA-Seq analysis under moderate O_3_ (75 ppb) and charcoal-filtered air (< 10 ppb) conditions. Ozone response was characterized for three leaf development positions to determine effects of leaf age.

## Results

### Response of genetically diverse wild soybean accessions to ozone

Previous analyses of publically available marker data identified 66 wild soybean accessions that capture most of the genetic diversity in the USDA wild soybean germplasm collections in maturity groups that are relevant to the Southern US (Song, personal communication, 2014). The response of 66 wild soybean accessions to 75 ppb O_3_ was determined by visually rating on a scale of 1 to 4 the characteristic foliar lesions following 5 days of O_3_ exposure (Table 1). Average ratings as low as 1.36 and as high as 4 were observed. PI 424123 and PI 507656 were chosen to represent the O_3_ resistant accessions (R) because of the low variability of the ratings among replications. PI 424007 and PI 407179 were chosen as representatives of the O_3_ sensitive (S) accessions because sufficient seed was readily available.

**Table 1:** Average ozone damage ratings (1-4) for a selected USDA *G. soja* diversity panel. Average represents the averaged leaf injury score, and SE represents the associated standard error of the measurement for 3 replications. Lower values indicate resistance, and higher values indicate sensitivity

Accession	Ozone Rating	SE
PI424116	1.36	0.36
PI245331	1.63	0.38
PI424123	1.75	0.25
PI507656	2.13	0.13
PI562551	2.25	0.50
PI378690	2.38	0.13
PI407240	2.38	0.38
PI407096	2.63	1.38
PI369621	2.63	0.13
PI339871A	2.77	0.23
PI562561	2.88	0.63
PI424025B	2.88	0.88
PI639588B	2.88	0.38
PI407038	3.00	0.50
PI407228	3.00	0.50
PI562553	3.00	0.50
PI424102A	3.00	0.25
PI407287	3.13	0.38
PI407300	3.13	0.38
PI407314	3.13	0.13
PI407085	3.53	0.29
PI424035	3.60	-
PI507761	3.13	0.13
PI562547	3.13	0.13
PI378696B	3.13	0.38
PI507581	3.25	0.25
PI507618	3.25	0.25
PI378684B	3.25	0.50
PI424070B	3.25	0.75
PI549046	3.25	0.26
PI407214	3.38	0.63
PI407248	3.38	0.13
PI378686B	3.38	0.13
PI378697A	3.38	0.13
PI597448D	3.38	0.38
PI507624	3.50	0.25
PI522226	3.50	0.50
PI549032	3.50	0.25
PI424004B	3.73	0.07
PI424082	3.61	0.17
PI407059	3.63	0.13
PI407157	3.63	0.13
PI407206	3.63	0.13
PI522209B	3.63	0.13
PI597460A	3.63	0.13
PI407195	3.70	0.14
PI407156	3.75	0.25
PI407020	3.75	0.25
PI424007	3.75	0.25
PI479752	3.75	0.25
PI522233	3.75	0.25
PI342622A	3.75	0.25
PI424083A	3.75	0.00
PI464890B	3.75	0.25
PI407179	3.88	0.13
PI407191	3.88	0.13
PI424045	3.88	0.13
PI447003A	3.88	0.13
PI522235B	3.88	0.13
PI366122	4.00	0.00
PI407231	4.00	0.00
PI468916	4.00	0.00
PI479768	4.00	0.00
PI483466	4.00	0.00
PI639623A	4.00	0.00
PI479746B	4.00	0.00

### Analysis of RNA transcripts from selected ozone sensitive and resistant wild soybean lines

Ninety-five samples were collected from four biological replicates of the first three fully expanded trifoliates (T1-T3) from both O_3_-treated (75 ppb) and charcoal-filtered (CF) air treated plants of PI 424007 (S), PI 407179 (S), PI 424123 (R) and PI 507656 (R). T1 represents the most mature leaf and T3 represents the least mature, though fully-expanded, trifoliate. Total RNA was isolated from leaf tissue for use in RNA-Seq analyses.

**Fig. 1:**
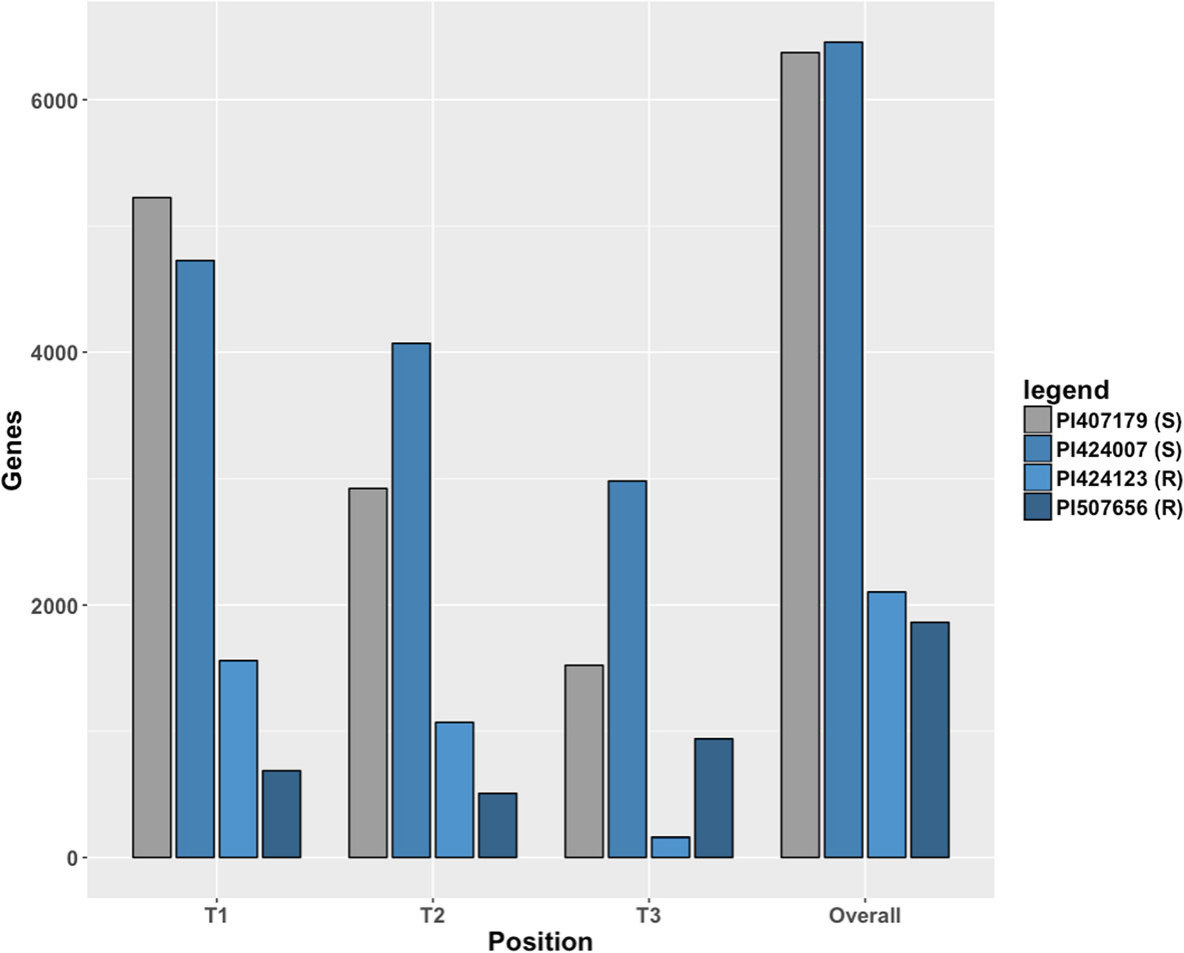
Differentially expressed genes by leaf position. Differential expression of genes in selected *G. soja* genotypes following ozone exposure. The histograms shows the number of genes responding in both directions at least 2-fold (FDR ≤ 0.05) between ozone treated and the control in the genotypes from each of the three leaf positions collected (T1, T2 and T3) as well as the total (overall) number in each genotype accounting for genes expressed in multiple leaves

The RNA-Seq data was analyzed by *cuffdiff* [22] to identify differentially-expressed genes. Comparisons were made between genotypes, treatments, and leaf positions. The numbers of genes that were differentially expressed following treatment with O_3_ relative to the CF control were identified and evaluated. In these studies, differential expression of genes was used to refer to changes in the steady state level of gene transcripts measured by RNA-Seq at which there was a change of at least 2-fold and a false discovery rate (FDR) of ≤0.05. While the exact numbers of genes differentially expressed varied by genotype, the two resistant genotypes, PI 424123 (R) and PI 507656 (R), differentially regulated similar numbers of genes at 2100 and 1863, respectively. Analogously, the number of genes responding in the two sensitive genotypes had similar responses to ozone: PI 407179 (S) had 6374 differentially-expressed genes and PI 424007 (S) had 6457 differentially-expressed genes (Fig 1).

**Fig. 2:**
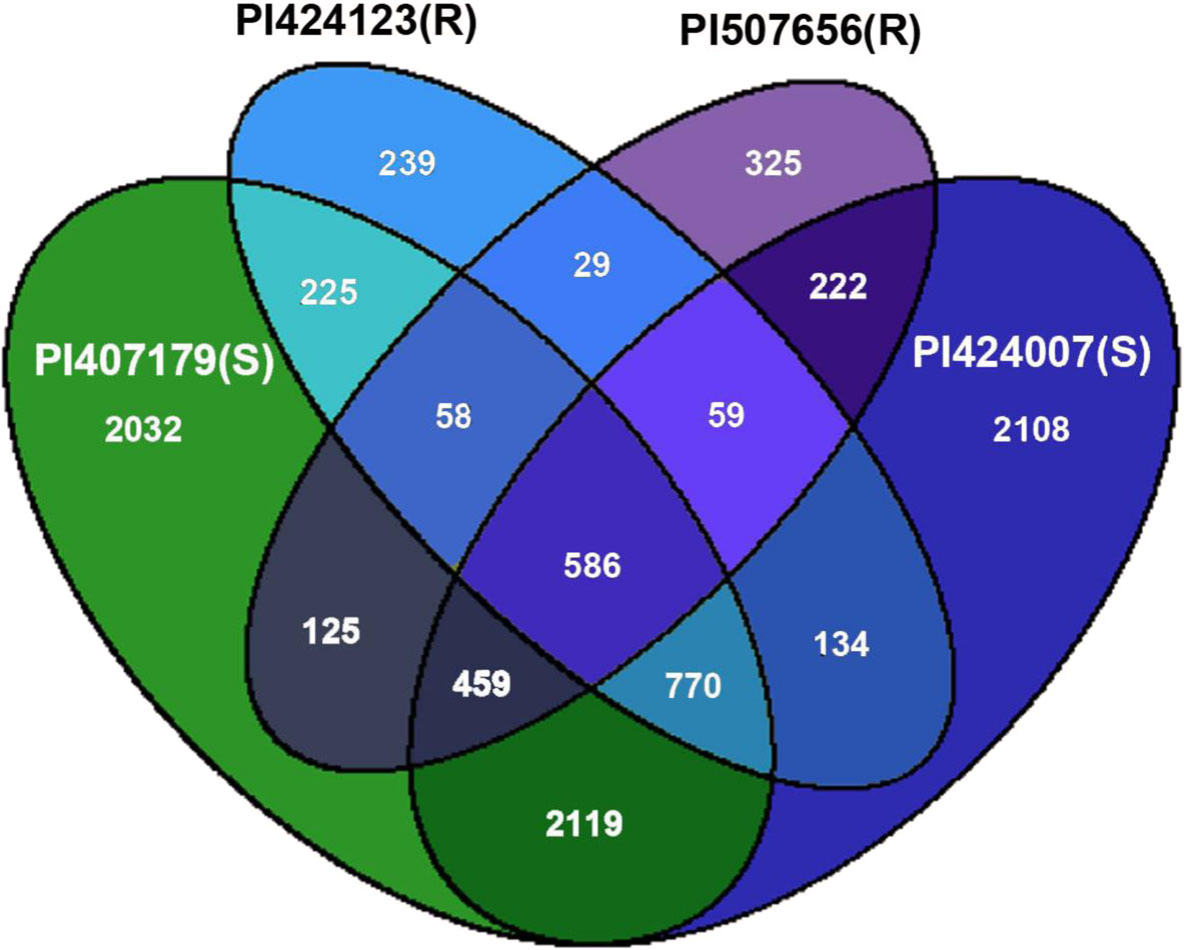
Venn diagram showing comparison of number of genes differentially expressed between accessions used in this study

The similarity of the genotypic responses to O_3_ was assessed by comparing the number of genes differentially regulated in response to O_3_ among accessions in any leaf position. Comparison of all genes differentially expressed in the resistant genotypes showed that the two genotypes shared responses for 732 (29 + 59 + 58 + 586) genes, (Fig 2). The sensitive accessions shared 3934 (586 + 770 + 459 + 2119) differentially-expressed genes in response to O_3_.

**Fig. 3:**
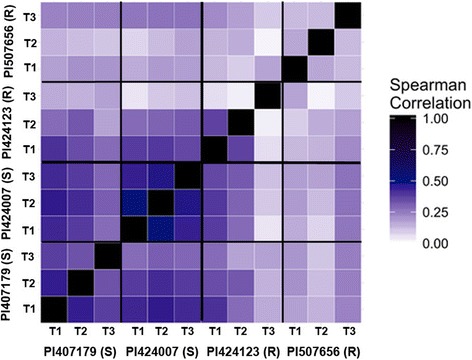
Heat map of correlations between gene expressions of leaf positions (T1-T3) in O_3_-sensitive accessions (PI 407179 and PI 424007) and O_3_-resistant accessions (PI 424123 and PI 507656)

Intra-genotypic Spearman correlations of gene expression levels between all combinations of expression data were obtained to characterize plant response to O_3_ based on age of leaf tissue. PI 424007 (S) displayed the most similar gene expression following O_3_ exposure between leaf positions with values from 0.44 -0.54; PI 407179 (S) was also highly correlated with values from 0.38 - 0.52. PI 424123 (R) showed similarity between the first two trifoliates (0.37), but neither T1 nor T2 had a correlation with T3 (0.08; 0.01, respectively). Correlations between leaf positions for PI 507656 (R) were more similar but were lower than those seen among sensitive genotypes (0.13 - 0.24) (Fig. 3).

Spearman correlations of the log2-fold change values for corresponding genes following ozone treatment were obtained to assess similarity of changes in gene expression in different leaf positions between accessions with the same response to ozone. These revealed changes in gene expression in response to ozone at different leaf positions that were more closely related in certain genotype/leaf position combinations than others (Fig. 3). For example, sensitive genotypes had a correlation of gene expression of 0.29 at the third trifoliate, 0.46 at the second trifoliate, and 0.43 at the first trifoliate, which was comparable to intra-genotype correlations. In contrast, gene expression of the resistant genotypes were less correlated at every position with 0.12 at the third trifoliate, 0.17 at the second trifoliate, and 0.19 at the first trifoliate.

### Ozone response varies in genotype based on leaf position

**Fig. 4:**
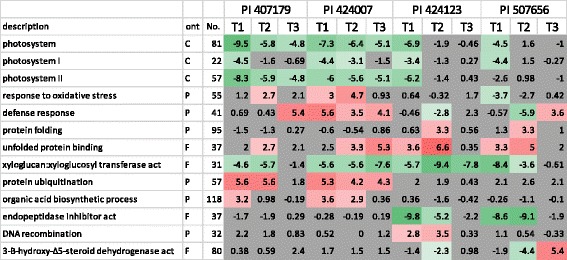
Selected gene ontologies enriched (*red*) or depleted (*green*) in leaves of different ages (T1 most mature to T3 least mature). Cells in *gray* were not statistically significant (q > 0.05). PI 407179 and PI 424007 are sensitive to ozone. PI 424123 and PI 507565 resistant to ozone. Description and Ont refers to the ontology enriched or depleted as determined by the PAGE tool at Agrigo. No. is the number of genes in the ontology

Parametric Analyses of Gene Set Enrichment (PAGE) were performed on the expression data to identify ontologies enriched or depleted in response to O_3_ exposure to gain insight into the effects these changes may have on leaf physiology [23]. Figure 4 reports the Z-score comparing enrichment between the target and reference sets of selected ontologies affected by O_3_ exposure, the complete list including *p*-values and expression means is available in Additional file 1. The Z-score is a value used in hypothesis testing that in this case combines the direction of change and the confidence in that change based on the variance of the ontology assuming a normal distribution at the selected FDR = 0.05 [23, 24]. As expected given the high correlations of gene expression among leaf positions in PI 424007 (S), many of the ontologies affected by O_3_ exposure were fairly consistent across leaf position (Fig. 4). We consider a response consistent if the Z-score holds significance. Changes in the average expression of the genes in a selected ontology were 2-fold or greater in the expected direction. However, the change in gene expression does not always reflect the amplitude of the Z-score. For example, the Z-scores of the “cell wall” ontology in T1 and T2 of PI 407179 (S) are −4.6 and −6.2, respectively, but average fold changes in expression of the genes in the ontology are both about −1.3 (Data not shown). We also note that the number of genes reported in Fig. 4 is the count of genes significantly expressed in any tissue tested, but differentially-expressed ontologies in a specific leaf may not include all of these genes. For example, the “cell wall” ontology in T1 and T2 of PI 407179 (S) are based on 34 and 29 genes (out of 57), respectively. Overall, the Z-score captures important effects of O_3_ treatment on gene expression given these caveats. Ontologies enriched/depleted in response to O_3_ that were consistent across all leaf ages in both O_3_ sensitive accessions include “Photosystem” and “Photosystem II”. Leaf T3 among the sensitive accessions was quite variable. Ozone effects between T1 and T2 compared among sensitive accessions shared ontologies of “protein ubiquitination”, “xyloglucan:xyloglucosyl transferase activity”, “cell wall” and “unfolded protein binding.” Other ontologies such as “photosystem I”, “response to oxidative stress”, “defense response”, “extracellular region” and “organic acid biosynthetic process” were enriched/depleted in PI 407179 (S) in the same direction as PI 424007 (S), but these were not consistent across leaf position. Differential expressions of genes associated with the ontologies “photosystem”, “photosystem I”, “protein folding”, “unfolded protein binding” and “endopeptidase inhibitor activity” had similar patterns of enrichment/depletion across leaf positions in both O_3_ resistant accessions (Fig. 4). Many other responses to O_3_ were leaf age specific.

### Comparison of resistant and sensitive genotype gene expression response to ozone

The most important goal of this research was to identify changes in gene expression associated with resistance to O_3_. Therefore, the transcriptional responses that contrast between sensitive and resistant accessions are the most likely to explain the phenotypic differences in the response of these accessions to O_3_ **.** Resistant genotypes appeared to down regulate “endopeptidase inhibitors” and “defense response” related genes in response to O_3_ unlike sensitive accessions (Fig. 4). Regulation of protease inhibitors has been associated with the defense response previously [25]. Genes related to “defense response” are uniquely depleted in resistant accessions. Genes associated with oxidative stress and defense were regulated in opposite directions in some leaf positions between O_3_ sensitive and resistant accessions (Fig. 4). Genes associated with “protein ubiquitination” and “organic acid biosynthesis” that were enriched in expression in O_3_ sensitive accessions were not significantly changed in resistant accessions. However, there was a leaf age specific response of the “protein folding” in response to O_3_ in the resistant accessions that contrasted with the sensitive accessions. Some changes occurred that were unique to specific resistant accessions such as enrichment/depletion of “DNA recombination” and/or “steroid dehydrogenase activity”. Genes associated with photosystem II were substantially depleted in the sensitive accessions in response to O_3_, but were only depleted in T1 of PI 424123 (R).

## Discussion

### Ozone resistance in the wild soybean germplasm

To our knowledge this is the first systematic screen for O_3_ tolerance in the *Glycine soja* germplasm. To maximize the chance of finding novel diversity for O_3_ tolerance we selected a group of wild accessions that are maximally genetically diverse. Wild soybean exposed to elevated O_3_ (75 ppb) had a range of responses from a rating of 1.36 to 4 (on a scale of 1-4). Overall these accessions were rather sensitive to O_3_ with average rating of 3.3 out of 4. However, 7 accessions scored less than 2.5 and 3 accessions scored less than 2. These accessions represent potential sources of O_3_-tolerance genes.

### RNA-Seq gene expression in wild soybean in response to ozone

To investigate the effects of O_3_ on gene expression in sensitive and resistant wild soybean accessions, we provided a treatment that could occur today during a moderate episode of elevated O_3_. There was a clear trend of fewer genes responding in younger leaves. This is a trend that has been noted before by Burton et al. [21] in a QTL-study for ozone response in cultivated soybean. However, this trend is not demonstrated for the O_3_ resistant accession PI 507656 (R). A parsimonious explanation for this different response to O_3_ is that the third trifoliate was at a slightly different stage of development in this particular accession, possibly related to genotypic differences among these diverse accessions. However, we note that T3 from PI 507656 (R) is no more correlated with other leaf positions than PI 424123 (R), and the correlation of T3 with other leaf positions was much lower in the O_3_-resistant accessions than the sensitive accessions. Therefore, it does not appear that the response of T3 in PI 507656 (R) can be attributed to leaf developmental stage and that there is a clear position specific effect of leaf age on response of the transcriptome in PI 507656 (R) that is not shared by any of the other accessions tested.

Many more genes responded to O_3_ exposure in sensitive genotypes than in resistant genotypes. Ozone resistance in these selected accessions is associated with a diminished plant response at the transcriptome level rather than a larger response to O_3_ (Fig. 4). Genes associated with “oxidative stress”, “defense response”, “protein ubiquitination” and “organic acid biosynthetic process” were uniquely regulated in the sensitive accessions compared to the resistant accessions. These are likely to be downstream effects of O_3_ exposure that are avoided in the O_3_-resistant accessions. For example, increased protein ubiquitination may indicate a turnover of protein in response to ROS. There are similar responses that appear to contrast between O_3_-sensitive and resistant accessions in an age specific manner. The most interesting of these age specific responses is that of “photosystem II”. Inhibition of photosynthesis is a well-studied effect of O_3_ exposure; the down-regulation of PSII-associated genes by a 1 day exposure to 75 ppb O_3_ may indicate that this aspect of photosynthesis is particularly vulnerable to elevated O_3_ in soybean. The responses of both resistant accessions appear to be less severe. However, T1 in PI 424123 (R) has a significant reduction in PSII-related gene expression. PSII-related gene expression was relatively unaffected by the O_3_ treatment in PI 507656 (R) and may indicate that photosynthetic capacity is preserved. This phenotype may help preserve crop yield in the face of rising O_3_ stress.

Apoplastic ROS quenching has been noted previously as a plant defense mechanism in response to pathogen attack and to other abiotic stressors including ozone [7, 26]. Additionally, up-regulation of xyloglucan endotransglucosylase/hydrolase genes has been linked to O_3_ damage in soybean previously [27]. Gene expression analysis of resistant and sensitive cultivated soybean (*G. max)* by Whaley et al. [21] revealed cultivar-specific responses to O_3_, and our study confirms a similar trend in soybean’s wild relative. Similar to the study in *G. max*, more genes responded in sensitive accessions versus resistant counterparts; however, due to the differences in experimental design, these studies were not directly comparable. Whaley et al. [21] noted an enrichment of wax and cutin biosynthetic genes and theorized that a difference in leaf anatomy could be responsible for response to O_3_; though, that same trend was not revealed from study in wild soybean. The differences between these studies could relate to cultivar-specific response mechanisms, the timing of leaf tissue harvests following O_3_ exposure, or the control level of O_3_ (27 ppb) used in that study compared to the CF treatment (<10 ppb) used in this experiment. While genes in ontologies related to the cell wall and extracellular region respond to O_3_, the study reported here failed to show differences in expression of genes related to the cell wall or extracellular spaces between the sensitive and resistant accessions of wild soybean.

A few responses to O_3_ were unique to the resistant accessions. Genes associated with protein folding are uniquely elevated in expression in T2 of both resistant accessions. We also note that a related ontology, the unfolded protein response, is generally elevated in T1 as well and is shared with O_3_ sensitive accessions. The increase in these ontologies in the resistant accessions suggests that they have greater capacity to deal with processing of unfolded proteins that arise as part of the stress response. This mechanism is likely to be different from the sensitive accessions given the difference in expression of genes associated with protein folding and protein ubiquitination between the sensitive and resistant accessions [28]. ROS are known to damage proteins, and pathways appear to be engaged to deal with this damage. Unfolded protein response (UPR), which describes the response of plants to improperly folded proteins, has been previously associated with plants responding to increased production of ROS [29]. Dealing with protein folding early in ROS exposure may help prevent downstream responses that lead to lesion formation and impair yield. Other changes in gene expression occurred that were unique to O_3_ resistant accessions. These changes targeted ontologies associated with endopeptidase activity, DNA recombination and steroid metabolism. The increased expression of genes associated with DNA recombination may be a downstream effect of O_3_ resistance or could simply reflect a poorly-annotated ontology. In some cases, we can reasonably speculate that these ontologies are related to the resistant phenotype. For example, reduction in the expression of endopeptidases may affect protein turnover or be related to plant defense. Steroid metabolism may be related to phytohormone production that is involved in a stress response.

## Conclusion

The wild soybean germplasm appears to be a valuable resource to improve tolerance to O_3_ in domesticated soybean. There are leaf-age specific responses both in the number of genes responding to O_3_ and the pathways targeted in response to O_3_. These position specific responses emphasize the importance of comparing similar tissues in gene expression analyses. Given the low correlation in the expression of genes responding to O_3_ between the two resistant accessions used in this study and differences between the pathways targeted by O_3_ treatment, both of these accessions might make unique contributions to improved O_3_ resistance in domesticated soybean. Wild soybean is a weedy relative of domesticated soybean that until recently was not considered a valuable resource for most soybean breeding programs. However, modern genomics combined with selection from large populations of progeny derived from crosses between *G. max* and *G. soja* has allowed the identification of a group of progeny that include most of the wild soybean genome in plants that do not share the viney architecture, seed shattering and hard seed coat of the wild parent. Indeed, agronomically-valuable progeny for 6 of the most O_3_-tolerant wild soybean accessions identified from this study are under development, and the heritability of O_3_ tolerance from the wild parent can be assessed soon.

## Methods

### Identification of phenotypic response of *Glycine soja* to increased ozone

Eight hundred six *G. soja* accessions from the USDA Soybean Germplasm Collection were identified as being >.01% genetically different than any other accession in the collection. These accessions were grouped into 81 clusters, and one accession from each cluster was chosen as being the most genetically diverse (Song, personal communication, 2014). Ozone responses for 66 of the 81 *Glycine soja* accessions chosen were evaluated in three annual trials from 2012 to 2014 (Table 1 **)**. The remaining 15 accessions were in early maturity groups that lacked relevance to Southern germplasm. Accessions were germinated and grown in a greenhouse with charcoal-filtered air (< 10 ppb O_3_) for a period of 14 days at the USDA-ARS facility in Raleigh, NC, USA. Plastic pots 15 cm in diameter filled with Fafard 2 Mix (Sungro, MA) and ~5 g of Osmocote Plus (Scotts Company, SC) were sown with 5 seeds but were thinned to 2 plants per pot after plants were established. One week after planting, Marathon (OHP, Inc., PA) (~1 g/pot) was applied for thrips prevention. The plants were then moved to continuous stirred tank reactors (CSTRs) in an adjacent greenhouse bay to acclimate to conditions 2 days prior to O_3_ exposure at 24 days after planting. Accessions were exposed in 3 replicated blocks in CSTRs subjected to a targeted elevated O_3_ treatment of 75 parts per billion (ppb) or a control treatment of charcoal filtered (CF) air for a period of 5 days. CSTRs are cylindrical chambers surrounded by Teflon film designed for containment and mixing of gases [30]. A cultivated soybean, Mandarin (Ottawa), identified as O_3_-sensitive from earlier *G. max* studies were included in the chambers for validation of treatment efficacy. Ozone damage on wild soybean is shown in Additional file 2.

The plants were rated by at least 5 individuals on a scale from 1 (resistant) to 4 (sensitive) based on percent foliar damage observed on mature leaves using a method adapted from Heagle [24] for characteristic O_3_ damage (1 = 0-5%, 2 = 5-25%, 3 = 25-50%, and 4 = 50-100%). Plants were rated alongside corresponding control treated plants (CF air) for verification of damage due to O_3_ and not some extraneous growth habit (Table 1 **)**. Compiling data from 2 years of phenotypic evaluation allowed for selection of two genotypes for gene expression study that consistently exhibit O_3_ resistance (PI 424123 and PI 507656) as well as two genotypes that were identified as sensitive to O_3_ (PI 424007 and PI 407179).

### Collection of ozone-treated samples

O_3_-sensitive wild soybean accessions PI 407179 (MG V) and PI 424007 (MG V) and O_3_-resistant wild soybean accessions PI 424123 (MG V) and PI 507656 (MG VII) were prepared for treatment with ozone as described previously. Individual trifoliates were collected separately at 4 PM following 7 h of treatment with 75 ppb O_3_. Each genotype had control plants in chambers under CF conditions. Trifoliates were also collected from these plants. Actual O_3_ levels were between 3 and 10 ppb for CF chambers and 75 ± 3 ppb for treatment chambers. CSTR temperature, relative humidity, and light levels were 35 ± 2 °C, 67 ± 6%, and 300 ± 60 μmol m^−2^ s^−1^ PAR, respectively, during the exposure period.

This experiment was replicated in 4 blocks within the greenhouse bay. Leaves from 2 plants of the same genotype within the same CSTR in each of two pots per chamber corresponding to the same trifoliate were pooled in order to obtain sufficient tissue for RNA isolation. The first three trifoliates (T1-T3) were collected from each plant. T1 was the most mature leaf, near the bottom of the plant, while T3 was the least mature leaf, near the top of the plant. Although less mature than T1 and T2, T3 was still a completely unfolded trifoliate in all plants. Samples were harvested into liquid nitrogen to prevent changes of gene expression during handling and to limit RNase activity. The samples were then transferred to a − 80 C freezer for storage.

### Isolation of RNA and sequencing

Total RNA was isolated using Plant RNeasy kits with on-column DNase I digestion (Qiagen, CA). Isolated samples were sent to the Genomic Sciences Laboratory (GSL) at North Carolina State University in Raleigh, NC where RNA quality was assured with Bioanalyzer RNA nano chips (Agilent, CA); Truseq libraries (Illumina, CA) were prepared; and libraries were sequenced using the HiSeq 2500 (Illumina, CA). Expressed transcripts for a total of 95 samples were sequenced (4 genotypes * 2 ozone conditions * 3 leaf positions * 4 replicates) – (1 degraded sample). Samples were multiplexed in lanes to allow for ~10 samples to be sequenced in each lane. Between 18 and 32 million × 125 bp single-end reads were obtained for each sample. Sequencing was done in 2 rounds of 2 replicates in August 2014 and the other 2 replicates in May 2015 for a total of four biological replicates for each sample.

### Analysis of RNA-Seq data

Samples were first analyzed for quality and nucleotide bias using the FastX-Toolkit (http://hannonlab.cshl.edu/fastx_toolkit/). The sequences were trimmed to 108 bases and quality-filtered to a PHRED score of 34. Trimmed sequences were then aligned to the Williams 82 soybean reference genome Wm82a2v1 with the available GFF file annotations using Tophat2 [31]. Alignment rates from the first set of sequencing were between 65 and 81%, while the second set aligned between 86 and 93% of input reads. In order to determine the cause of lower alignment for the first samples, alignment of the unmapped sequences from the first run of sequencing was run with the chloroplast and mitochondrial genomes. The results showed an additive alignment of 85-95%, indicating that lower genomic alignments were caused by a higher presence of chloroplastic and mitochondrial mRNA.

Differential expression was measured with Cufflinks 2 [22] using the Williams Wma2v1 reference genome annotations as a guide for transcript assembly. Gene expression is reported in Fragments Per Kilobase of transcript per Million mapped reads (FPKM). FPKM normalizes counts of short sequences by read depth and transcript length. Differential expression was determined using *cuffidff* output for genes exhibiting significance at a false discovery rate (FDR) of *p* < 0.05 coupled with a log2-fold change of ≥1 or ≤ −1. Recent work by the SEQC/MACQ-III Consortium [32] has displayed a strong correlation between expression data from RNA-Seq and qPCR (>80%), so qPCR validation was not performed on this data.

Significant differentially-expressed genes following O_3_ treatment were analyzed for associated gene ontologies (GO) using the Gene set enrichment analysis (GSEA) tool, called PAGE, on AgriGO [23]. For each sample, the GSEA results returned GO term categories enriched or depleted in response to O_3_ found in each sample based on a false discover rate (FDR) < 0.05. The Bonferroni correction option was used. Using available gene model annotation information, gene numbers reported could fit multiple ontology categories. Ontology categories in this study were reduced to eliminate redundancy of identical gene sets matching similar categories. These were chosen to match the most detailed descriptions without presenting misleading gene numbers. Expression analysis was visualized with graphs and figures created using *CummeRbund*, an R package for data visualization of Cufflinks data [33], ggplot2 [34] and the *VennDiagram* R packages [35]. Sequence reads, as well as the full listing of genes responding differentially between treatments with associated FPKM and log2-fold changes, can be found on the NCBI Gene Expression Omnibus (Project: GSE85146) (http://www.ncbi.nlm.nih.gov/geo/).

## Additional files

Additional file 1: The complete list of *p* values and Z scores of ontologies affected by O_3._ Included are the GO accessions, the category of the GO, a description of the GO, the number of genes in the GO, Z scores and *P* values for all comparisons between wild soybean accessions treated with CF and O_3_. (XLSX 270 kb)

Additional file 2: Typical ozone damage on wild soybean. O_3_ damage is shown on PI 468396 A and PI 504287 A. (DOCX 549 kb)

## Abbreviations

CF: Charcoal-filtered
CSTR: Continuously stirred tank reactors
FDR: False discovery rate
FPKM: Fragments Per Kilobase of transcript per Million mapped reads
GFF: General feature format
GO: Gene ontology
MAPK: Mitogen-activated protein kinase
O_3_: Ozone
PI: Plant introduction
Ppb: Parts per billion
PS: Photosystem
R: O_3_ resistant
ROS: Reactive oxygen species
S: O_3_ sensitive
T1: First fully expanded trifoliate
T2: Second fully expanded trifoliate
T3: Third fully expanded trifoliate
UPR: Unfolded protein response
