# Supplementary material for: RNA-Seq study reveals genetic responses of diverse wild soybean accessions to increased ozone levels
Source: BMC Genomics. 2017 Jun 29;18:498. doi: 10.1186/s12864-017-3876-2 (PMC5493002; doi:10.1186/s12864-017-3876-2)
Supplement: Supplementary file 2 — Typical ozone damage on wild soybean. O3 damage is shown on PI 468396 A and PI 504287 A. (DOCX 549 kb) [file 12864_2017_3876_MOESM2_ESM.docx]

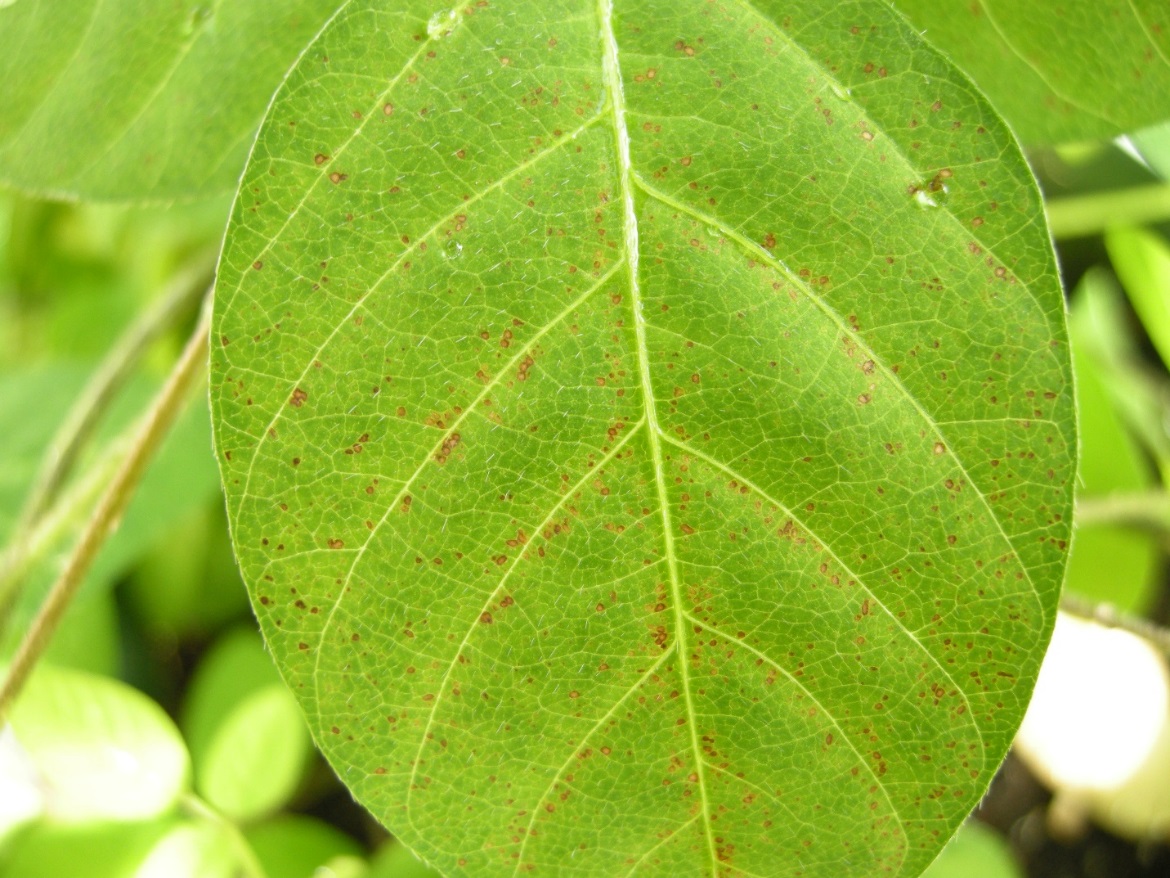


PI 468396 A


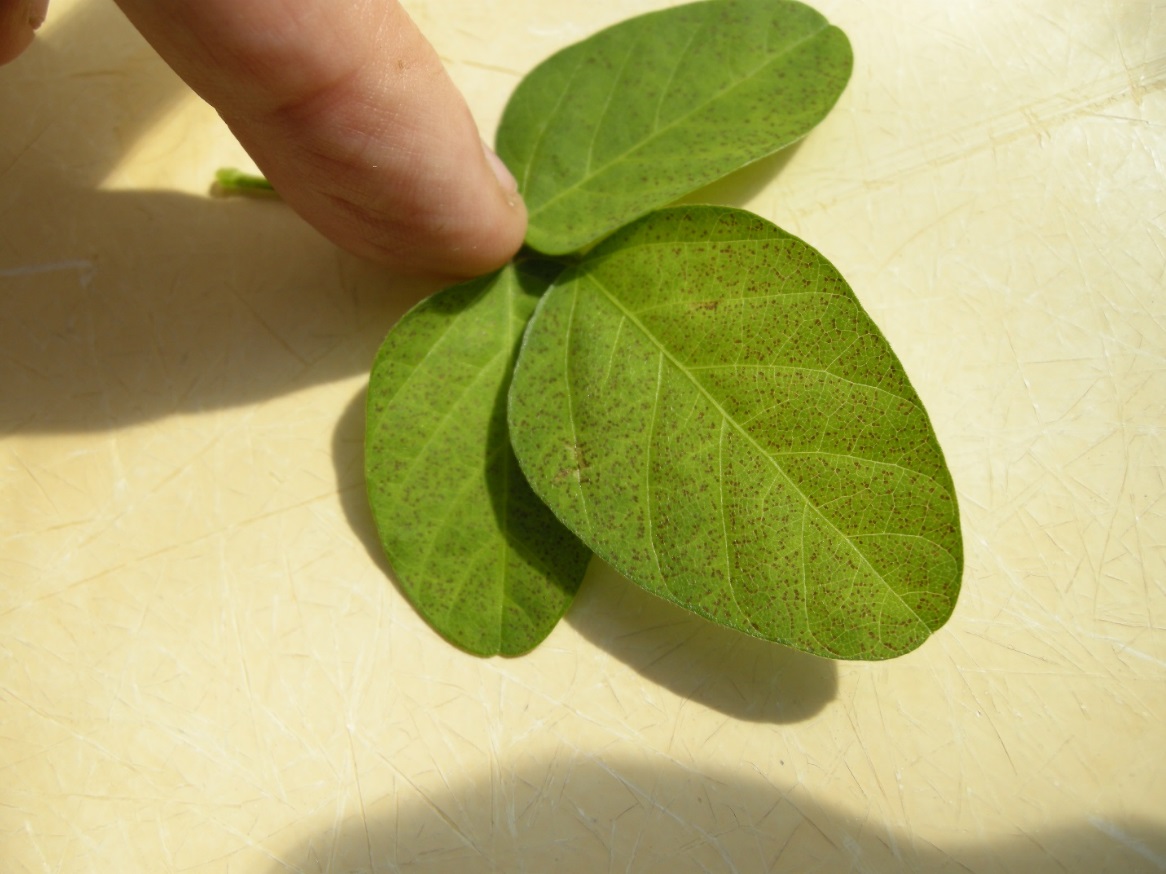


PI 504287 A

These images illustrated the damaged caused by a 5 day exposure to elevated ozone on two sensitive accessions of wild soybean. The lesions on PI 468396A or associated with veins while the lesions on PI 504287A are in interveinal regions also well. Note that these images illustrate that the lesions in response to ozone are similar to domesticated soybean but that were used to rate ozone damage but were not part of the actual experiment used to rate ozone damage of the core set of wild soybean.
